# Parallel bioreactor system for accessible and reproducible anaerobic culture

**DOI:** 10.1099/acmi.0.000225

**Published:** 2021-04-15

**Authors:** Taylor I. Monaghan, Joseph A. Baker, Gary K. Robinson, Mark Shepherd

**Affiliations:** ^1^​ School of Biosciences, RAPID Group, University of Kent, Canterbury, CT2 7NJ, UK

**Keywords:** anaerobic, *Clostridium*, ABE fermentation, bioreactor

## Abstract

When working with anaerobic bacteria it is important to have the capability to perform parallel bioreactor growth experiments that are both controllable and reproducible, although capital and consumables costs for commercially available systems are often prohibitively high. Hence, a three-vessel parallel bioreactor system was designed and constructed that has the capabilities for batch and fed batch processes and can also be set up for continuous culture at a fraction of the cost of commercial systems. This system carries over many of the same functionalities of those systems with a higher price point of entry, including in-line monitoring of temperature, pH, and redox poise. To validate the performance of this system *Clostridium saccharoperbutylacetonicum* was grown under conditions that promote ABE fermentation, an established industrial process used to produce the solvents acetone, butanol and ethanol. Measurements of cell density, pH, and redox poise all confirmed reproducible culture conditions for these parallel vessels, and solvent quantitation via GCMS verified consistent metabolic activities for the separate cultures. In future, this system will be of interest to researchers that require high performance parallel fermentation platforms but where commercial systems are not accessible.

## Introduction

One of the main hurdles in establishing a fermentation protocol is the cost of entry per unit. Prices for these fermentation units can range from entry at £8000 per unit, to upwards of £25000 per unit. Furthermore, parallel fermenter systems with disposable vessels are both expensive to instal and come with notoriously high consumable costs. Hence there is a need for a fermentation system that retains the functionality of commercially available units but at a fraction of their cost. In terms of retaining the main functionalities of commercial systems, these would be variable fermentation mode (i.e. batch, fed batch or continuous culture) and compatibility with variable culture volumes and a variety of probes/electrodes.

The test system chosen to assess the bioreactor system developed herein was ABE fermentation by clostridial species, an established process for industrial butanol production dating back over a century [1]. In brief, this growth physiology is described by two distinct phases, the first being acidogenesis where acetic and butyric acid are produced, followed by solventogenesis where these acids are re-assimilated to form acetone, butanol and ethanol. Different clostridial species are able to utilise a large variety of carbon sources, from C1 gases [2] and simple mono- and disaccharides [3], through to complex polysaccharides such as cellulose and hemicellulose [4, 5], making them ideal candidates to metabolise a large variety of waste material and providing a sustainable and environmentally friendly bioprocess. Some clostridia can also grow autotrophically by fixing CO_2_ and CO chemolithotrophically converting them to acetyl-CoA for solvent production [6]. While this may be useful in an industrial setting to recycle waste gases, the current study required an anaerobic organism that was easy to grow heterotrophically. Hence, *Clostridium saccharoperbutylacetonicum* NI-4(HMT) [7] was chosen for this growth study as it grows well on 5C (e.g. xylose) and 6C (e.g. glucose) sugars and is relatively aerotolerant.

For the culture of solventogenic clostridial strains three main types of fermentation are commonly used: batch, fed batch and continuous fermentations. Fed-batch systems are usually run initially as a simple batch fermentation to allow for the establishment of a dense culture medium for entry into the solventogenic phase. Once this phase is reached, the cells are fed with additional feedstock to extend the solventogenic phase to increase the solvent yield. However, due to the toxicity of solvent accumulation, butanol in particular, fed-batch systems have traditionally been investigated in tandem with various solvent recovery methods to maximise the solvent production and yield [8–11]. Continuous fermentation takes place in a chemostat where the concentrations of cells, substrates and products are at a steady state following the gradual addition of multiple culture volumes of fresh fermentation broth. One of the main drawbacks of continuous fermentation with *Clostridium* is a low cell count. This has been overcome via the use of cell immobilisation in various carriers as well as cell recycling using membranes [12], although this would seem unnecessarily complicated for the current study. Finally, batch fermentations use an initial high concentration of feedstock that is catabolised for the production of ABE solvents, and is often carried out using very simple anaerobic vessels without the capability monitoring of pH or redox poise. Indeed, *C. saccharoperbutylacetonicum* has been grown in this way using various feedstocks: mix of glucose/butyric acid [13]; wastewater algal biomass [14]; sago starch [4]; mix of cellobiose and xylose to eliminate catabolite repression [15]. The monitoring and control of pH is crucial during batch ABE fermentation as the drop of pH during acid production enables the solventogenic shift to occur [16]. However, the pH cannot be allowed to drop too far and cause acid crash (i.e. <pH 5) and the pH must not become too alkaline as this will lead to inefficient solvent production [17]. In addition, for efficient conversion of acids to solvents, a significant amount of reducing power is required (e.g. in the form of NADH or other reducing agents), which is reflected in the redox poise of the growth medium. Hence, along with temperature regulation, it is also desirable to incorporate pH and redox electrodes into anoxic bioreactors used for solventogenic clostridia. Of the three techniques batch culture is the simplest mode of growth and is clearly an established method for ABE fermentation. Hence, the batch method was chosen as the technique to showcase the hardware and methods developed in the current study. Herein, we describe a new method for inexpensive anaerobic fermentation that is robust, easy to sterilise, adaptable in terms of in-line monitoring, and can be used to generate highly reproducible metabolite data.

## Methods

### Bacterial isolates

*Clostridium saccharoperbutylacetonicum* N1-4(HMT) (DSM-14923/ATCC 27021) was used for this study. Reinforced clostridium medium (RCM) (Sigma 27546–500 G-F) was made anaerobic by autoclaving at 121 °C at 15 psi for 20 min in serum bottles sealed with butyl stoppers. Depending upon the starter culture needed for an experiment either 30 ml or 100 ml of RCM was inoculated with 500 µl of 15 % (v/v) glycerol stocks of *C. saccharoperbutylacetonicum* N1-4(HMT) and grown anaerobically in sealed serum bottles at 32 °C.

### Batch fermentations

Batch fermentation was carried out in 1 litre culture vessels (SciLabware, Pyrex Quickfit) with 500 ml culture volumes at 32 °C. Fermentation medium consisted of yeast extract tryptone medium (YETM) (40 g l^−1^ glucose, 2.5 g l^−1^ yeast extract, 2.5 g l^−1^ tryptone, 0.5 g l^−1^ ammonium sulphate and 0.025 g l^−1^ iron sulphate) at pH 6.2, supplemented with 0.1 M 2-(N-morpholino)ethanesulfonic acid (MES) free acid (Merck) for pH control. MES buffer was chosen as it has a pK_a_ of 6 and has previously been shown to be very effective at the pH range observed during ABE fermentation [18]. To generate an alternative feedstock with more viscous cellulosic properties, apple pomace was produced from whole apples blended to a slurry and strained through muslin cloth. This pomace was mixed with distilled water to give a pomace-water mixture comprised of 10 % w/v pomace, and the pH of the suspension was adjusted to 7.0 with NaOH. Anaerobic conditions in the fermentation were generated by sparging filtered (0.2 µm pore size) oxygen-free nitrogen through the fermentation medium for 20 min pre-inoculation and then 5 min post-inoculation. Seed cultures were established by growing recovered RCM grown cells in 80 ml YETM in serum bottles overnight to an OD_600_ of ~4.0. The final inoculation was 10 % (v/v). Fermentations were carried out in triplicate. Throughout the fermentations OD_600_, pH and redox poise (Mettler Toldeo, InLab Redox Micro) of the fermentation medium was measured. The redox electrode was calibrated against quinhydrone (87 mV at pH 7.0, 264 mV at pH 4.0, Normal Hydrogen Electrode (NHE) correction for a Ag/AgCl electrode in 3M KCl =+210 mV). Following sampling, supernatant and cells were separated by centrifugation at 8000 ***g*** for 10 min. Supernatant and cell pellet were separated and frozen at −80 °C for later use.

### Acid and solvent quantitation

Acids and solvents from culture supernatants were quantitated using Gas Chromatography Mass Spectrometry (GCMS) using an Agilent 6890 N instrument. The GC was equipped with a Phenomenex 7HG-6013–11 Zebron column. Helium (>99.999 %) was used as the carrier gas, with a constant flow rate of 1 ml min^−1^. A 0.2 µl water sample was injected with a 100 : 1 split ratio (i.e. a common parameter that describes proportion of sample that is transferred to the column). Injection temperature was set to 150 °C, the GCMS transfer line temperature was set to 280 °C, ion source to 230 °C, and quadrapole to 150 °C. After injection, column temperature was held at 30 °C for 5 min, after which this increased at a linear gradient to 150 °C at the 20 min mark. Compounds were identified by comparison of retention times with those of reference standards. Example chromatograms and standard curves are shown in Figs S1 and S2 and (available in the online version of this article), respectively.

### Sugar quantitation

Culture supernatants were homogenised and centrifuged at 13400 ***g*** for 5 min. Then 200 µl of the sample was then added to 600 µl of HPLC grade water, achieving a ×4 dilution and a total volume of 800 µl. Glucose concentrations were measured using cation exchange chromatography at 60 °C using a phenomenex rezex ROA H+column at 1 ml min^−1^ 5 mM sulphuric acid using an Agilent 1100 series refractive index detector to monitor glucose elution. Concentrations of samples were determined by comparison to a standard curve for glucose with integrated peak areas used for the determination of glucose concentration (Fig. S3).

## Results

### Bioreactor design and construction

**Fig. 1.**
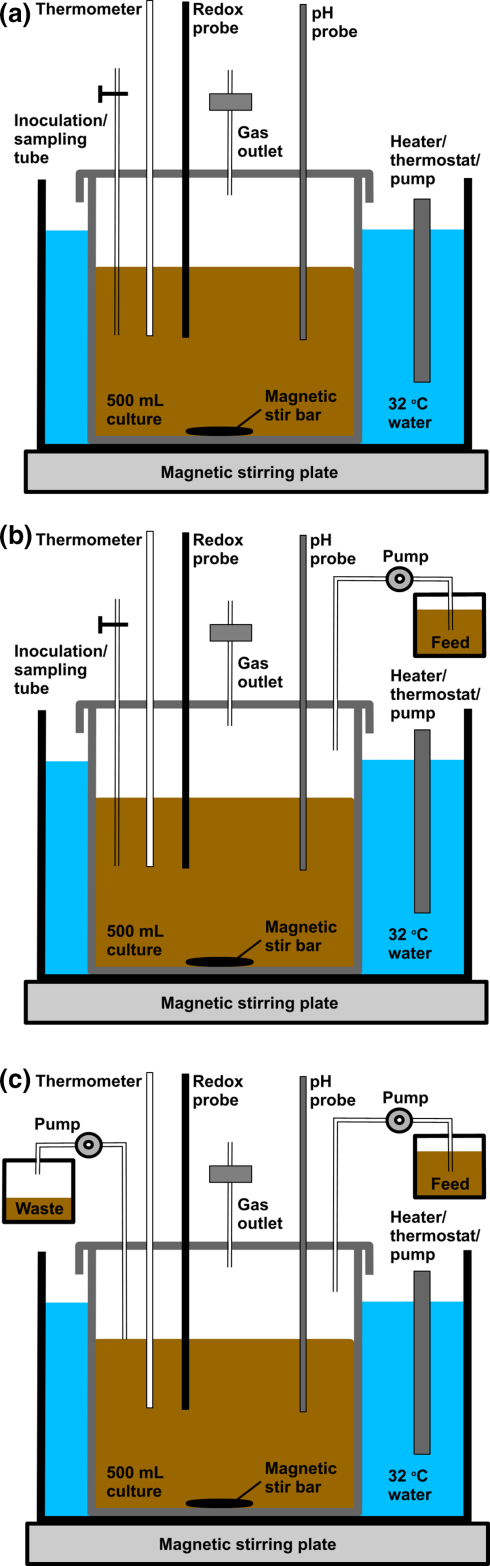
Schematic diagrams of the anoxic bioreactor units. (**a**) Batch fermentation system. (**b**) Fed batch. (**c**) Continuous culture.

To identify required functionalities for anoxic bioreactor systems, commercially available fermentation systems were assessed via consulting technical specifications obtained from company websites and sales representatives, and schematics for a new fermentation system to be used in batch, fed batch as well as continuous setup were drawn up (Fig. 1). From here, a list of components to be purchased was created (Table 1), and a custom gas escape/sample port had to be constructed by running metal piping at varying lengths through rubber bungs and sealing them with rubber bungs. In future, this outlet could be linked up to ‘add on’ analytical instrumentation should the user be interested in measuring, for example, nitrogen gases or calculating mass balances.

**Table 1.** Components used in designed fermentation system. All components that are necessary for the construction of the vessel are included in the list. Peristaltic pumps for fed-batch and continuous culture are not costed as these are optional extras for most bioreactor systems

Component	Brand and model
One litre culture vessel	SciLabware. FV1L Quickfit 1LT culture vessel 100 mm flat flange
Culture vessel port lid	SciLabware. MAF4/41 Quickfit lid 100 mm flat flange 3X sockets 14/23 and 2x socket
Culture vessel gasket	SciLabware. PS100 100 mm flat flange PTFE seal
Lid to vessel clips	SciLabware JC100F Quickfit joint clips metal, spring wire, FG 100 (for EX5/105)
Suba seals	Fischer Scientific. Stopper turnover flange and serrations rubber white 19 mm plug diameter
0.2 µm gas filters	Whatman. Polydisc TF Chemical resistant in-line filter
Aquarium heater and thermometer	U-picks Aquarium Heater (Amazon)
Water pump	Maxesla Submersible Pump (Amazon)
pH probe	Mettler Toledo. pH electrode InLab Semi-Micro-L
Redox probe	Mettler Toledo. ORP electrode InLab Redox-L
Magnetic stirrer	Scientific Laboratory Supplies

**Fig. 2.**
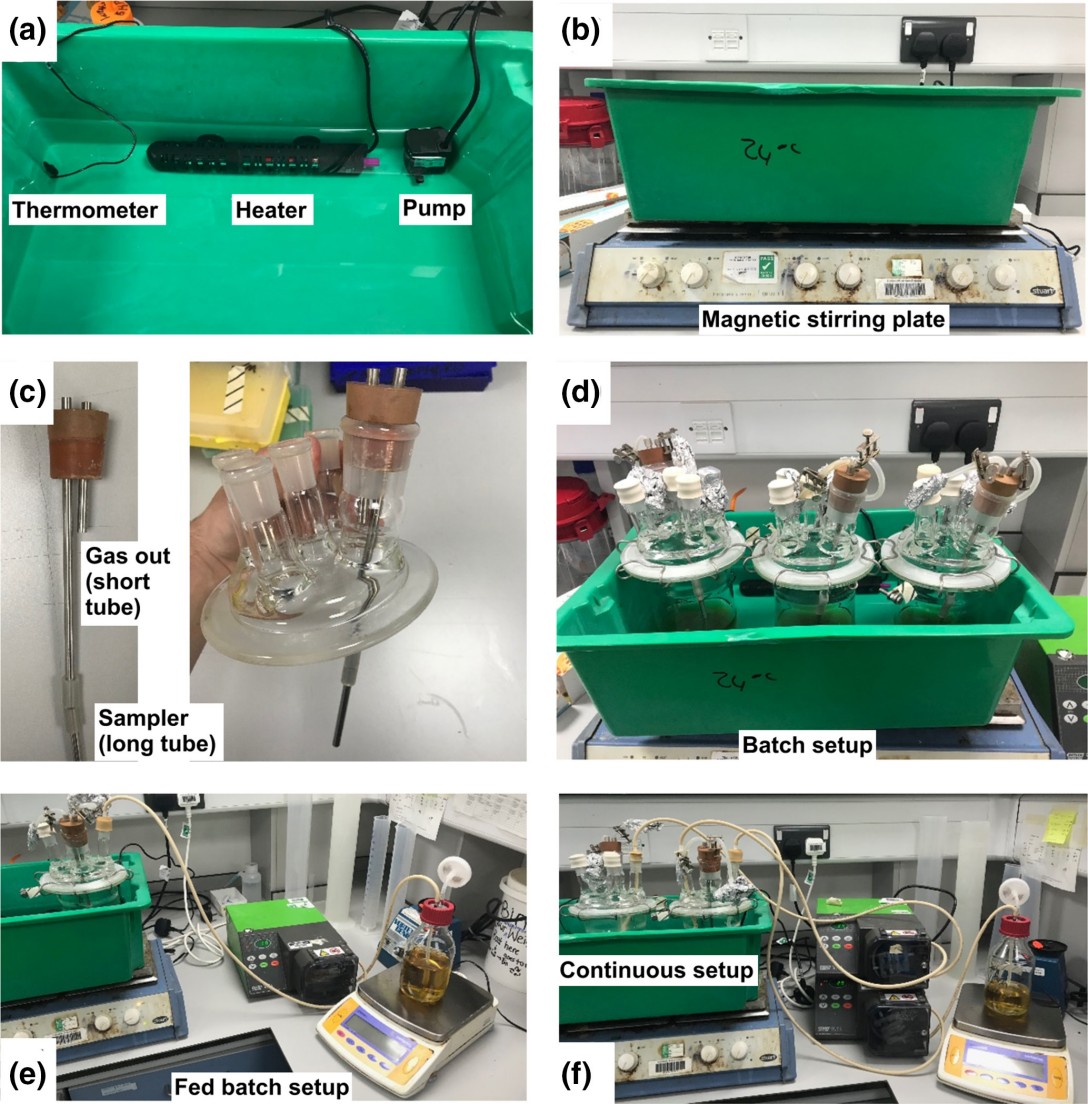
Components and final setup of the anoxic bioreactor. (**a**) Temperature control system. (**b**) Mode of agitation. (**c**) Gas outlet and sampling tubes. (**d**) Three parallel bioreactors in batch mode. (**e**) Fed-batch mode. (**f**) Continuous culture mode.

Most commercial systems use either an electric heated jacket system, such as the Fermac 200 (Electrolab) and the F0-Baby (Bionet), or a jacketed water system like that seen on the BioFlo 120 (Eppendorf). A water jacketed system was chosen where the temperature was controlled in the range 18–30 °C by using a budget aquarium heater (a Sous Vide machine may also work well for this). Alongside temperature control, culture agitation was achieved via the use of a magnetic stirring bead with the growth vessels placed on magnetic stirrers. Redox and pH measurements were achieved via the use of the Mettler Toledo InLab electrodes attached to a hand-held monitor. Images of the final setup can be seen in Fig. 2, and functionality and cost are compared with commercially available systems in Table 2. All of these systems can be setup to run in fed-batch or continuous mode, which requires an additional pump. For the current system a conventional peristaltic pump was used, which are available to most research labs and can be purchased for as little as £1000.

**Table 2.** Comparison of main functionality of commercially available fermentation systems compared to the current system

System				
	Electrolab FerMac 200	Eppendorf BioFlo 120	BioNet F0-BABY	Current system
**In-Line Functionality**				
Temperature control range (°C)	5–50	0–70	Temp Range not given	18–37
pH control range	4–10	2–12	2–12	Controlled via buffering agent
Agitation (rpm)	50–1100	25–1500	0–2000	0–1000
In-line OD measurements	Yes - Optional Extra	No	No	No
Redox (mV)	Yes – optional probe	Yes	Yes - optional probe	Yes
Dissolved O_2_	0–120 %	0 200%	Yes – Optional extra	No – optional extra.
**Fermentation type**				
Batch	Yes	Yes	Yes	Yes
Fed batch	Yes – optional extra pump	Yes – built in	Yes – optional extra pump	Yes – optional extra pump
Continuous	Yes – optional extra pump	Yes – built in	Yes – optional extra pump	Yes – optional extra pump
Volume range (L)	2–1000	0.25–40	1–5	1
**Price per unit (GBP**)	8000	20 000	19 995	2150

### Monitoring growth conditions and metabolite analysis for ABE fermentation

**Fig. 3.**
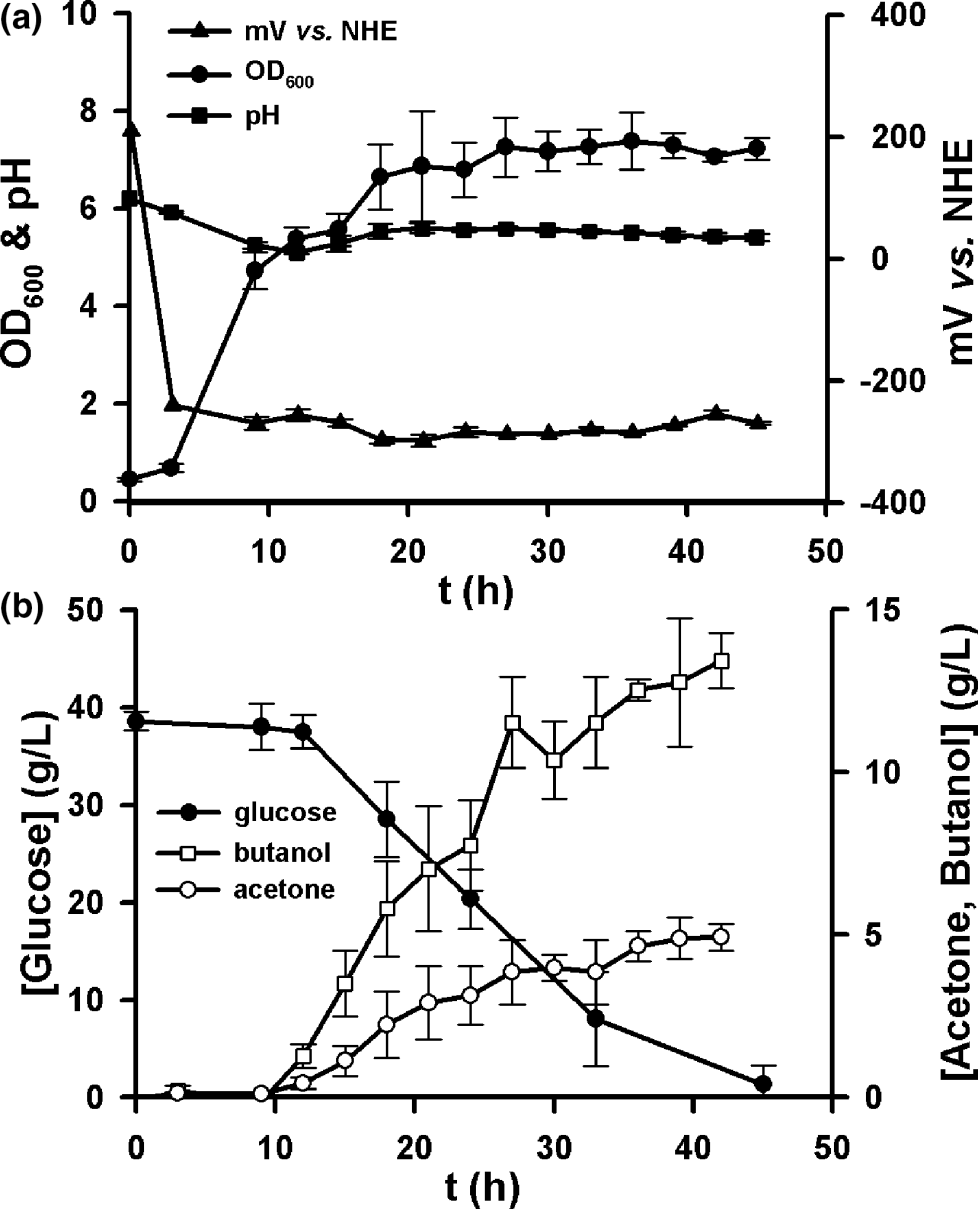
Monitoring of growth parameters and metabolites for a *C. saccharoperbutylacetonicum* N1-4(HMT) batch fermentation. (**a**) Output obtained for OD_600,_ pH and redox (mV vs. Normal Hydrogen Electrode (NHE)). (**b**) Glucose consumption during fermentation measured using HPLC, and evolution of acetone and butanol. Fermentations were performed in triplicate and error bars show standard deviation.

The bioreactor system was initially trialled in batch, fed-batch, and continuous mode: batch mode was chosen to showcase this system due to ease of comparison with previous ABE fermentation studies. Parallel batch fermentations were undertaken with 500 ml cultures of wild-type *C. saccharoperbutylacetonicum* N1-4(HMT) grown at 32 °C for 48 h. Anaerobic conditions were established by purging with filtered (0.2 µm pore) N_2_ for 30 min per vessel prior to inoculation and then 10 min following inoculation. The batch fermentations were run in triplicate and set up as illustrated by the schematics in Fig. 1. The final OD_600_ measured was 7.2±0.2, the lowest pH value reached was 5.09±0.03, and the lowest redox value reached was −298±9 mV vs. Normal Hydrogen Electrode (NHE) (Fig. 3a). Glucose consumption was measured using HPLC (Fig. 3b), demonstrating that this diminished from an initial 40 g l^−1^ to 1.3±2.0 g l^−1^. Solvent production started at 12 h and were at maximum levels at 45 h, with a peak acetone concentration of 4.9±0.5 g l^−1^ and a peak butanol concentration of 13.4±0.8 g l^−1^.

## Discussion

As the cost of specialist fermentation technology can be high (Table 2), the current project aimed to create a cost-effective alternative that uses affordable parts in conjunction with equipment available in most labs (e.g. spectrophotometer for OD_600_ readings). The system presented herein is versatile, easy to instal and run, and shares many of the same functionalities as current commercial models such as, pH and redox measurements, along with the ability to be set up for batch, fed batch or continuous processes (Figs 1 and 2). The current bioreactor system can be set up for less than a quarter of the cheapest commercially available unit, and for one tenth the cost of certain popular brands (Table 2).

In order to test the new anoxic bioreactor setup, ABE fermentation was chosen as it is an established industrial process with several parameters that can be measured to assess both growth conditions and metabolic activity. Cell densities were clearly highly reproducible across all three bioreactors (Fig. 3a), as were rates of glucose consumption (Fig. 3b). The in-line redox and pH electrodes were very useful to monitor the drop in pH and redox poise (Fig. 3a) that are expected during ABE fermentation, and the initial rise and fall in acid concentrations and subsequent conversion to solvents (acetone and butanol) can be clearly seen in Fig. 3c. The final titre of butanol was a reproducible 13 g l^−1^, which is a healthy concentration considering that maximal levels plateau at approximately 20 g l^−1^ due to the membrane toxicity of butanol [19, 20]. Indeed, this titre is comparable to published butanol titres for *C. sacchararoperbutylacetonicum* cultures grown using commercially available fermenter systems (Table S1) [21–24]. To further investigate the use of this fermentation setup with a more turbid and viscous growth medium (i.e. more common in industrial feedstocks), experiments were also undertaken using a 10 % apple pomace medium. While changes in optical density were not sensible to measure in this turbid medium, other growth parameters (pH and redox) were reproducibly recorded to track the progress of this experiment (Fig. S4). These data indicate that growth in this medium elicited a delayed pH shift and transition to ABE solventogenesis, resulting in lower but reproducible final concentrations of butanol and acetone. Given that no chemical/enzymatic upstream processing of the pomace was performed, it was encouraging that solventogenesis took place and we are confident that this feedstock can be optimised in future.

Without the need to maintain constant oxygen concentrations, the anaerobic bioreactor is a simple piece of equipment that does not require expensive components to create the ideal environment for anoxic growth (as has been demonstrated for ABE fermentation). While commercial systems may have additional in-line monitoring systems, the physical dimensions and mechanical operation are very similar to the current setup, and although it is anticipated that the majority of users will work with conventional growth media the current system can also cope with a viscous feedstock. To conclude, herein we have demonstrated that it is possible to design and construct a fermentation system that has similar functionality to commercial units at a fraction of the cost. This system can be set up and running within a week, is very easy to clean and autoclave, and offers a great deal more functionality for anoxic culture compared to serum bottles and anoxic jars. The current setup has also been used to culture clostridial cultures on a variety of growth media, and has proved to be a reliable workhorse to investigate upstream processing of industrial feedstocks for ABE fermentation. Clearly, the cost element is key which makes parallel growth vessels much more accessible. Hence, this work will be of interest to researchers aiming to perform highly reproducible anoxic growth experiments but the lack the resource to purchase multiple units from commercial outlets.

## Supplementary Data

Supplementary material 1
