# Supplementary material for: Parallel bioreactor system for accessible and reproducible anaerobic culture
Source: Access Microbiol. 2021 Apr 15;3(4):000225. doi: 10.1099/acmi.0.000225 (PMC8208757; doi:10.1099/acmi.0.000225)
Supplement: Supplementary material 1 [file acmi-3-0225-s001.pdf]

Supplemental Data

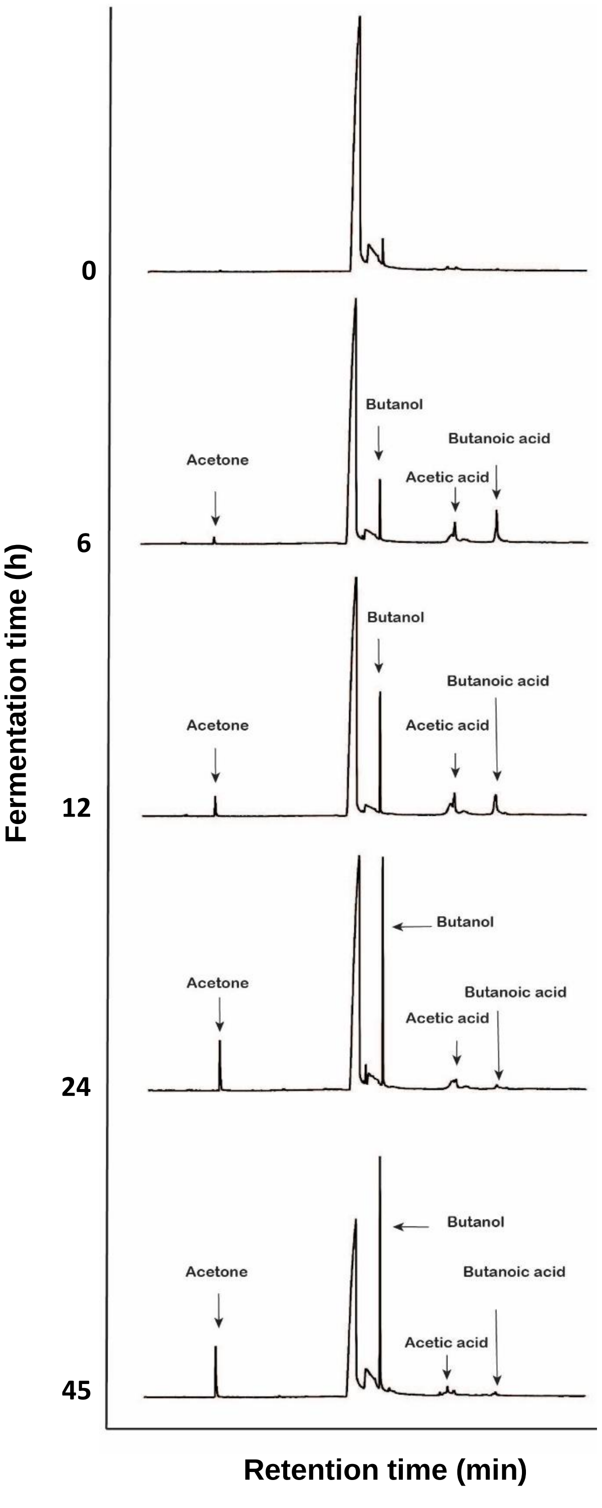

Fig. S1. GCMS Chromatographs from *C. saccharoperbutylacetonicum* N1-4(HMT) YETM 40 g L $^{-1}$ batch fermentation for the identification of acetone and butanol as well as acetic acid and butanoic acid. 2 mL of cell culture was taken from cells growing in a batch fermentation and cells were removed via centrifugation. Sample supernatants were applied to the GCMS instrument and peak positions were identified using known standards.

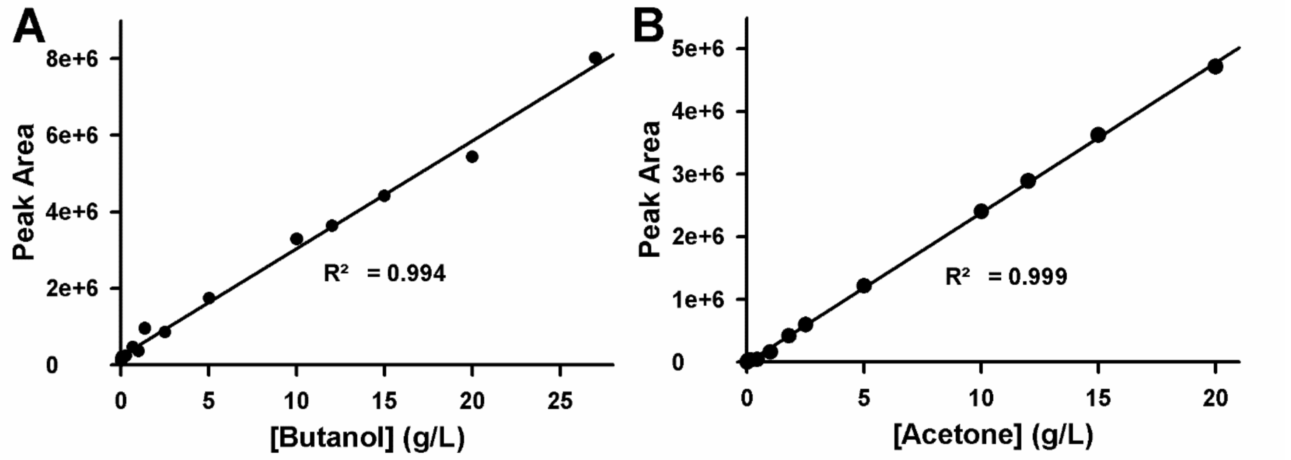

Fig. S2. GCMS standard curves. Solvents were diluted in water and were analysed via GCMS. A) A) Acetone. B) Butanol.

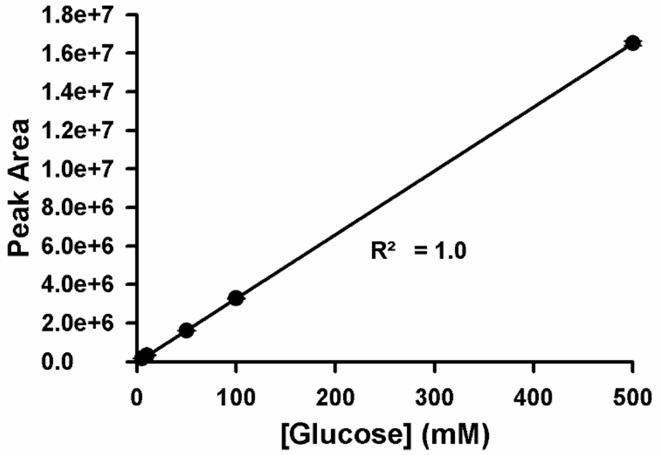

Fig. S3. Glucose standard curve. Glucose was quantitated via HPLC with refractive index detection as described in the Materials and Methods section. Measurements are averages of 3 repeats and error bars show standard deviations.

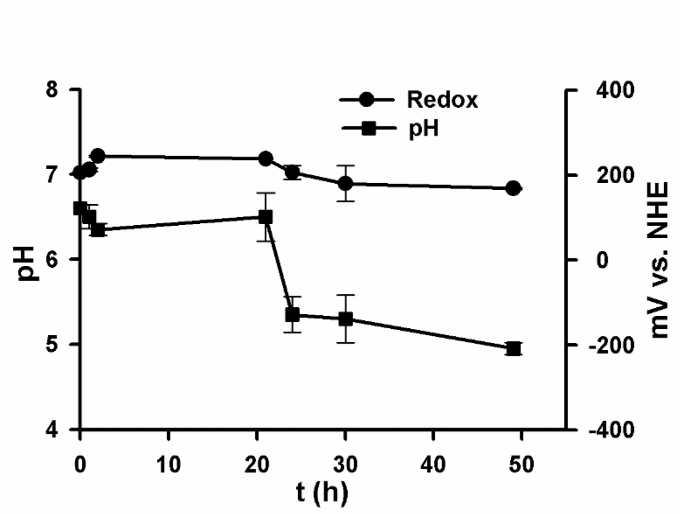

Fig. S4. Monitoring of growth parameters and metabolites for a *C. saccharoperbutylacetonicum* N1-4(HMT) batch fermentation on apple pomace. Output obtained for pH and redox (mV *vs.* Normal Hydrogen Electrode (NHE)). Final concentrations of butanol and acetone were measured as (0.6 ± 0.3 gL^{-1}) and (0.4 ± 0.03 gL^{-1}), respectively. Fermentations were performed in duplicate and error bars show standard deviation.

Fermenter	Feedstock	OD_{600}	pH control	Butanol (g/L)	Acetone (g/L)	Source
Current system	YETM + glucose	7.2	MES	13.4 ± 0.8	4.9 ± 0.5	This paper
Eppendorf BioFlo 120	P2 medium	15.3	NaOH	15.3 ± 0.1	6.7 ± 0.1	(21)
Eppendorf BioFlo 120	TGY + lactose	17.4	NaOH	17.4 ± 0.85	7.6 ± 0.53	(22)
Multifors 5L bioreactor (Infors HT)	PGY + P2 glucose(40 g /L)	2	Not controlled	9.3	3.4	(23)
LiFlus GX; Biotron 5L	CGM + glucose	10.0	Ammonia	17.6	4.4	(24)

Table S1. Comparison of current solvent yields with published ABE fermentation data. Cultures of solventogenic clostridia were grown in different growth media either in the current fermenters or a commercially available system. Abbreviations: CGM, clostridia growth medium; TGY, tryptone-glucose-yeast extract medium; YETM, yeast extract tryptone medium.
